# Antifungal Activity of Fibrate-Based Compounds and Substituted Pyrroles That Inhibit the Enzyme 3-Hydroxy-methyl-glutaryl-CoA Reductase of *Candida glabrata* (CgHMGR), Thus Decreasing Yeast Viability and Ergosterol Synthesis

**DOI:** 10.1128/spectrum.01642-21

**Published:** 2022-04-04

**Authors:** Damián A. Madrigal-Aguilar, Adilene Gonzalez-Silva, Blanca Rosales-Acosta, Celia Bautista-Crescencio, Jossué Ortiz-Álvarez, Carlos H. Escalante, Jaime Sánchez-Navarrete, César Hernández-Rodríguez, Germán Chamorro-Cevallos, Joaquín Tamariz, Lourdes Villa-Tanaca

**Affiliations:** a Departamento de Química Orgánica, Escuela Nacional de Ciencias Biológicas, Instituto Politécnico Nacional, Mexico City, Mexico; b Departamento de Microbiología, Escuela Nacional de Ciencias Biológicas, Instituto Politécnico Nacional, Mexico City, Mexico; c Laboratorio de Investigación Microbiológica, Hospital Juárez de México, Mexico City, Mexico; d Departamento de Farmacia, Escuela Nacional de Ciencias Biológicas, Instituto Politécnico Nacional, Mexico City, Mexico; Mycology Laboratory, Wadsworth Center

**Keywords:** HMGR, ergosterol, fibrates, pyrroles, atorvastatin, synthetic antifungal, *Candida*, multidrug resistance

## Abstract

Due to the emergence of multidrug-resistant strains of yeasts belonging to the Candida genus, there is an urgent need to discover antifungal agents directed at alternative molecular targets. The aim of the current study was to evaluate the capacity of three different series of synthetic compounds to inhibit the Candida glabrata enzyme denominated 3-hydroxy-methyl-glutaryl-CoA reductase and thus affect ergosterol synthesis and yeast viability. Compounds 1c (α-asarone-related) and 5b (with a pyrrolic core) were selected as the best antifungal candidates among over 20 synthetic compounds studied. Both inhibited the growth of fluconazole-resistant and fluconazole-susceptible C. glabrata strains. A yeast growth rescue experiment based on the addition of exogenous ergosterol showed that the compounds act by inhibiting the mevalonate synthesis pathway. A greater recovery of yeast growth occurred for the C. glabrata 43 fluconazole-resistant (versus fluconazole-susceptible) strain and after treatment with 1c (versus 5b). Given that the compounds decreased the concentration of ergosterol in the yeast strains, they probably target ergosterol synthesis. According to the docking analysis, the inhibitory effect of 1c and 5b could possibly be mediated by their interaction with the amino acid residues of the catalytic site of the enzyme. Since 1c displayed higher binding energy than α-asarone and 5b, it is the best candidate for further research, which should include structural modifications to increase its specificity and potency. The derivatives could then be examined with *in vivo* animal models using a therapeutic dose.

**IMPORTANCE** Within the context of the COVID-19 pandemic, there is currently an epidemiological alert in health care services due to outbreaks of Candida auris, Candida glabrata, and other fungal species multiresistant to conventional antifungals. Therefore, it is important to propose alternative molecular targets, as well as new antifungals. The three series of synthetic compounds herein designed and synthesized are inhibitors of ergosterol synthesis in yeasts. Of the more than 20 compounds studied, two were selected as the best antifungal candidates. These compounds were able to inhibit the growth and synthesis of ergosterol in C. glabrata strains, whether susceptible or resistant to fluconazole. The rational design of antifungal compounds derived from clinical drugs (statins, fibrates, etc.) has many advantages. Future studies are needed to modify the structure of the two present test compounds to obtain safer and less toxic antifungals. Moreover, it is important to carry out a more in-depth mechanistic approach.

## INTRODUCTION

The emergence of multidrug-resistant strains of Candida yeasts in recent years has made infections by these pathogens a more serious problem (1). Although Candida albicans, C. glabrata, Candida tropicalis, Candida parapsilosis, and Candida krusei are species isolated from healthy individuals, they can behave as invasive opportunistic pathogens under host conditions of a compromised immune system.

Among the particularly important Candida species with multidrug resistance are Candida auris, the species of the Candida haemulonii complex, and C. glabrata. They cause in-hospital outbreaks and polymicrobial infections associated with SARS-COV-2 (2, 3). C. glabrata is intrinsically resistant to azoles, and its recent pan-echinocandin-resistant strains are also associated with the COVID-19 pandemic (4). This strain has been proposed as a model for the study of statins as antifungal agents (5).

To date, three main mechanisms of antifungal action have been found for antifungal agents, involving alterations of the fungal membrane by the binding of polyenes to ergosterol; of the synthesis of ergosterol by the activity of azoles, allylamines, and thiocarbamates; and of the generation of the cell wall by echinocandins (6). A possible alternative target is 3-hydroxy-methyl-glutaryl-CoA (HMGR), an enzyme that catalyzes the synthesis of mevalonate, one of the critical steps in the ergosterol biosynthesis pathway (7–10). The purpose of developing new antifungals with alternative molecular targets is to provide a wide range of compounds capable of responding to the multidrug resistance of Candida spp. and other fungi.

The aim of the present study was to evaluate the capacity of new synthetic compounds to inhibit the C. glabrata HMGR enzyme (CgHMGR) and therefore affect ergosterol synthesis and yeast viability. Two series of compounds were derived from fibrate-based acyl- and alkyl-phenoxyacetic methyl esters, as well as 1,2-dihydroquinolines (11). A third series was developed from substituted pyrroles (12, 13). The best compound in each series was subjected to *in vitro* experiments to assess yeast growth, the level of ergosterol, and yeast growth rescue with the addition of exogenous ergosterol. The experimental data were complemented with docking simulations.

## RESULTS

### Selection of the best CgHMGR inhibitors.

**FIG 1:**
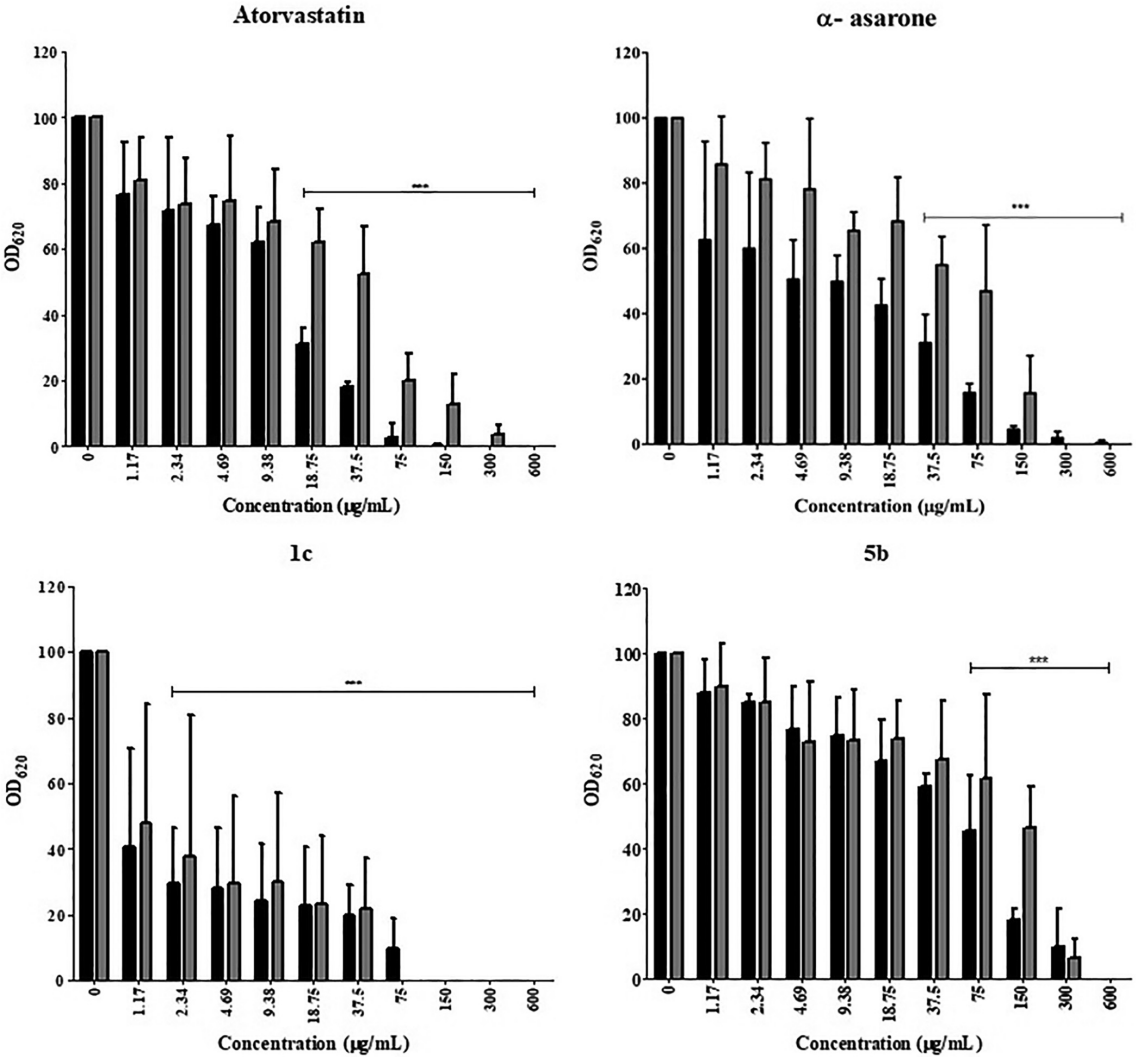
Inhibition of the growth of C. glabrata CBS 138 (black bars) and C. glabrata 43 (gray bars) by HMGR inhibitors (antifungal reference and test compounds). As a control, the strains were grown without any inhibitor. The optical density (OD) was determined in a Thermo Scientific Multiskan FC microplate photometer at 620 nm (OD_620_) after incubation for 24 h at 37°C. The quantification of yeast growth was based on OD values, which were expressed as the average of three independent assays ± SD. Significant differences were analyzed by two-way analysis of variance (ANOVA). ***, *P* < 0.001.

An evaluation was made of the possible antifungal activity of the 13 compounds of series 1 and 2 and the seven compounds of series 3. The controls were the dimethyl sulfoxide (DMSO) solvent and two compounds (α-asarone and fluconazole, at different concentrations) that reduce the synthesis of ergosterol in C. glabrata (Fig. S1; Fig. 1). The best inhibition of the growth of C. glabrata in solid YPD medium was exhibited by derivative 1c (of series 1 and 2) and the substituted pyrrole derivate 5b (of series 3) (Fig. S1; Fig. 1).

### The HMGR inhibitors affect the viability of C. glabrata.

**FIG 2:**
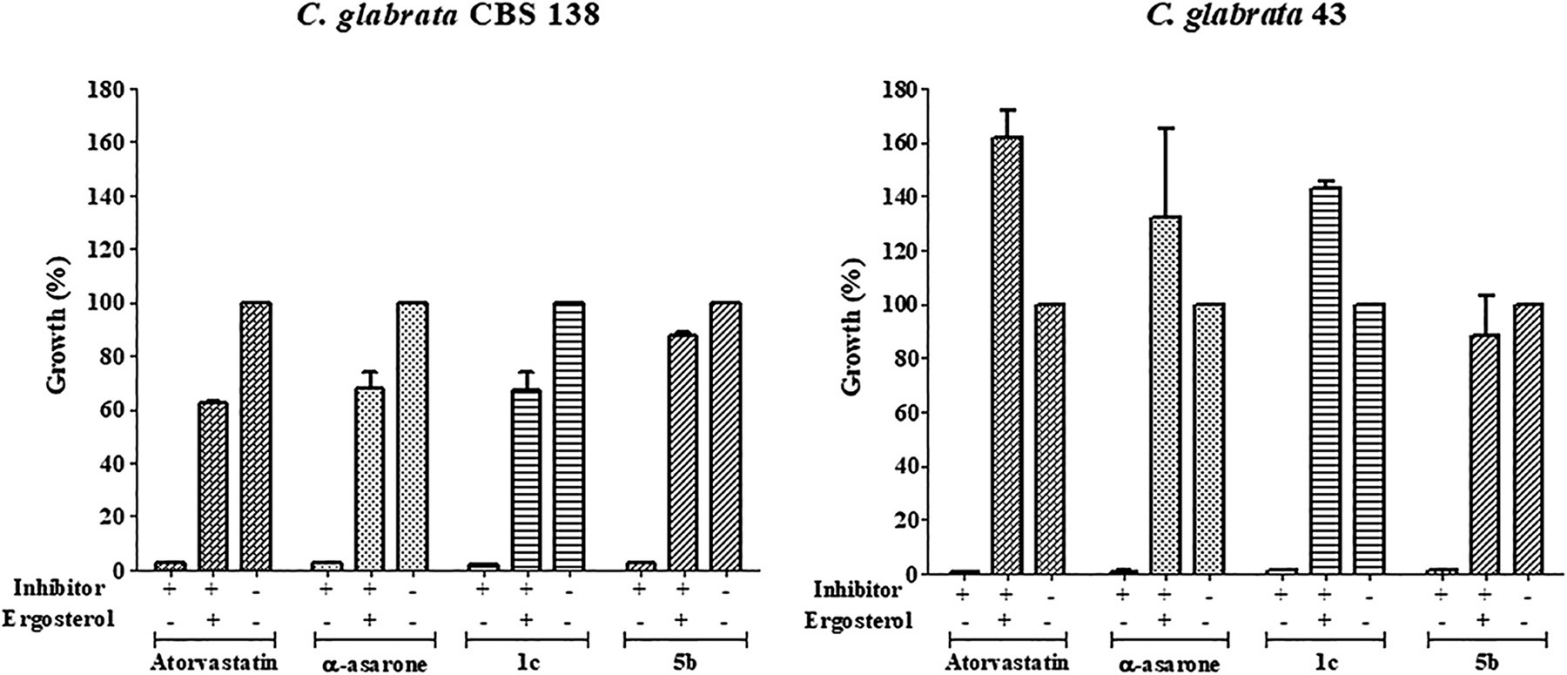
Yeast growth rescue experiment of C. glabrata with ergosterol. After yeast growth was stopped by treatment with HMGR inhibitors (antifungal reference and test compounds used at their IC_70–90_), the addition of exogenous ergosterol led to a recovery of the growth of C. glabrata CBS 138 and C. glabrata 43. As a control, the strains were also grown without any inhibitor (considered 100% growth). + represents addition of the inhibitor or ergosterol to the medium; – indicates the absence of the same. After incubation for 24 h at 37°C, the optical density was determined in a Thermo Scientific Multiskan FC microplate photometer at 620 nm. Growth rate values (As_620_) are expressed as the average of three independent assays ± SD. ***, *P* < 0.001 compared to the assay without any inhibitor, based on the Student’s *t* test.

The phenotype of the strains was verified: C. glabrata CBS 138 and C. glabrata 43, which are susceptible and resistant to fluconazole, respectively. Once this was established, an evaluation was made of the *in vitro* antifungal activity of 1c, 5b, α-asarone (to which 1c is structurally related), and atorvastatin (an HMGR inhibitor to which 5b is structurally related). Both the test (1c and 5b) and reference compounds (α-asarone and atorvastatin) were able to diminish the viability of the two strains of C. glabrata. Compound 1c at 75 µg/mL provided growth inhibition similar to atorvastatin and α-asarone at the same concentration, reducing yeast growth by up to 90% for the two strains. It was necessary to apply 300 µg/mL of 5b to afford a similar percentage of inhibition (Fig. 1 and 2). As the concentration of the compound increased, the growth of the yeast strains decreased (Tables 1 and 2), indicating a dose-response effect. Compound 1c presented lower IC_50_ and IC_70–90_ values than its control (α-asarone), 5b, and atorvastatin (Table 3).

**TABLE 1:** Effect of 1c, 5b, α-asarone, and atorvastatin on the growth of C. glabrata CBS 138

Inhibitor concn (µg/mL)	Inhibition (% of relative growth ± SD)^*a*^			
	Atorvastatin	α-Asarone	1c	5b
0	0	0	0	0
1.17	23.4 ± 15.8	37.4 ± 30.2	59.4 ± 29.9	12.3 ± 10.1
2.34	28.5 ± 22.6	40 ± 23.3	70.6 ± 16.9***	15.4 ± 2.5
4.69	32.5 ± 8.3	49.5 ± 12.1	71.9 ± 18.2***	23.4 ± 13.1
9.38	38 ± 10.6	50.2 ± 8.0	75.9 ± 17.5***	25.7 ± 11.7
18.75	68.9 ± 5.0***	57.4 ± 8.1	77.3 ± 18.2***	33.3 ± 12.5
37.5	81.8 ± 1.1***	68.9 ± 8.7***	80.1 ± 9.3***	41.0 ± 3.9
75	97.5 ± 4.2***	84.2 ± 2.8***	90.4 ± 9.1***	54.6 ± 17.1***
150	100 ± 0.4***	95.5 ± 1.1***	100 ± 0***	81.8 ± 3.4***
300	100 ± 0***	98.06 ± 1.9***	100 ± 0***	90.1 ± 11.3***
600	100 ± 0***	99.51 ± 0.8***	100 ± 0***	100 ± 0***

a The relative growth was calculated as a percentage of the growth detected in the absence of any inhibitor (considered 100%). The original results were obtained by the optical density, determined in a Thermo Scientific Multiskan FC microplate photometer at 620 nm (OD_620_), after incubation for 24 h at 37°C. The data are expressed as the averages of three replicates ± SD. Significant differences were analyzed with two-way analysis of variance (ANOVA). ***, *P* < 0.001.

**TABLE 2:** Effect of 1c, 5b, α-asarone, and atorvastatin on the growth of C. glabrata 43

Inhibitor concn (µg/mL)	Inhibition (% of relative growth ± SD)^*a*^			
	Atorvastatin	α-Asarone	1c	5b
0	0	0	0	0
1.17	19.4 ± 13.4	14.2 ± 14.7	52.2 ± 36.5	10.7 ± 13.1
2.34	26.2 ± 13.7	18.9 ± 11.2	62.2 ± 42.7***	15.1 ± 13.7
4.69	25.7 ± 20.1	21.9 ± 21.6	70.3 ± 26.5***	27.2 ± 18.3
9.38	31.7 ± 15.8	34.6 ± 5.7	70.0 ± 27.1***	26.9 ± 15.5
18.75	38.0 ± 10.2***	31.7 ± 13.5	76.6 ± 20.7***	26.6 ± 11.7
37.5	47.8 ± 14.4***	45.1 ± 8.72***	77.9 ± 15.0***	32.6 ± 17.7
75	80.1 ± 8.3***	53.1 ± 20.3***	100 ± 0***	38.5 ± 25.7***
150	87.2 ± 8.9***	84.3 ± 11.5***	100 ± 0***	53.7 ± 12.6***
300	96.5 ± 3.0***	100 ± 0***	100 ± 0***	93.9 ± 6.2***
600	100 ± 0***	100 ± 0***	100 ± 0***	100 ± 0***

a The relative growth was calculated as a percentage of the growth detected in the absence of any inhibitor (considered 100%). The original results were obtained by the optical density, determined in a Thermo Scientific Multiskan FC microplate photometer at 620 nm (OD_620_), after incubation for 24 h at 37°C. The data are expressed as the averages of three replicates ± SD. Significant differences were analyzed with two-way ANOVA.

***, *P* < 0.001.

**TABLE 3:** MIC_50_ and MIC_70–90_ values of 1c, 5b, α-asarone, and atorvastatin against C. glabrata^*a*^

Inhibitor	C. glabrata CBS 138		C. glabrata 43	
	MIC_50_ (µg/mL)	MIC_70–90_ (µg/mL)	MIC_50_ (µg/mL)	MIC_70–90_ (µg/mL)
Control	—^*b*^	—	—	—
Atorvastatin	13	37.5	40.1	195.2
α-Asarone	9.38	113.5	60.5	204.5
1c	<1.17	75	<1.17	58
5b	62.3	300	131.7	108.2

a The control consisted of the yeast strain cultivated without any inhibitor.

b The dashes mean that the treatment was not applied to the control strains.

### For C. glabrata treated with inhibitors, growth recovered after adding ergosterol.

A yeast growth rescue experiment was carried out to verify that the inhibition of the HMGR enzyme affects the levels of ergosterol, the final product of the biosynthesis pathway (Fig. 2). The compounds were applied at the sublethal concentrations estimated in the previous experiment (minimal inhibitory concentration at 70 to 90% [MIC_70–90_]). When exogenous ergosterol was subsequently added to the culture medium, yeast growth did indeed occur, in contrast to the lack of growth caused by the inhibitor. In some cases, such as with 1c applied to C. glabrata 43, the recovery of yeast growth reached an even higher level than the control (the yeast cultured in the absence of an inhibitor). Thus, this finding confirmed that the compound derived from α-asarone altered the pathway for the production of ergosterol in C. glabrata and more specifically that it targeted the synthesis of the HMGR enzyme.

### The test compounds (CgHMGR inhibitors) affect ergosterol biosynthesis in C. glabrata.

**FIG 3:**
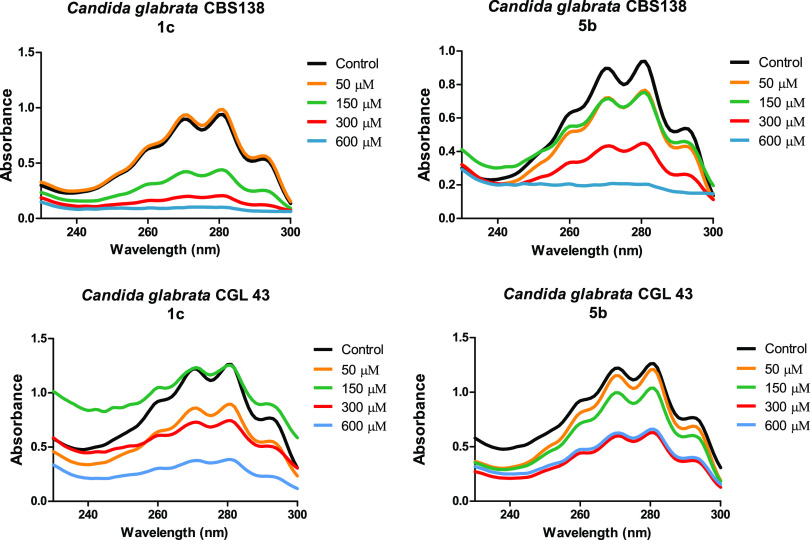
CgHMGR inhibitors 1c and 5b lowered the level of ergosterol. C. glabrata CBS138 and C. glabrata 43 were grown in YPD medium and treated with different concentrations (50, 100, 300, and 600 μM) of the inhibitors. The control was the YPD medium without any inhibitor or treated with the vehicle (dimethyl sulfoxide [DMSO]) only. For each treatment, the yeasts were incubated at 37°C for 18 h under constant shaking at 200 rpm. By spectrophotometrically scanning (from 230 to 300 nm) the extracted sterols (in the *n*-heptane layer), their presence, absence, or possible reduction could be detected.

To explore the possible association between the loss of viability of C. glabrata and the inhibition of the production of ergosterol, the level of ergosterol in the yeasts was measured after 18 h of treatment with 1c, 5b, simvastatin, or α-asarone (the latter two as reference compounds; data not shown). The corresponding absorption spectra (Fig. 3) contained the characteristic four peaks of sterols. The test compounds caused a reduction in the level of sterols in both the fluconazole-susceptible and -resistant strains of C. glabrata.

The absorption peak corresponding to 281.5 nm was used to quantify the concentration of ergosterol, allowing for the calculation of the percentage of inhibition of its synthesis (Table 4). In general, residual ergosterol levels were higher in the C. glabrata 43 versus C. glabrata CBS 138 strain. In both strains, a greater decrease in ergosterol was caused by 1c than 5b. Simvastatin and α-asarone served as positive controls for the inhibition of CgHMGR, since previous studies demonstrated their capability of inhibiting the recombinant HMGR of C. glabrata (8). The higher the concentration of the inhibitor, the greater was the percentage of inhibition of ergosterol synthesis (Table 4).

**TABLE 4:** Percentage of ergosterol inhibition of C. glabrata cells treated with HMGR enzyme inhibitors^*a*^

Inhibitor	Concn (μM)	C. glabrata CBS 138	C. glabrata 43
Control (W/I)	—^*b*^	100	100
DMSO control	—	100	100
Simvastatin	50	62.3	82.5
	150	19.6	79.9
	300	8.4	67.7
	600	7.9	54.8
α-Asarone	50	65.2	81.1
	150	36.3	60.4
	300	15.23	53.3
	600	0.00	23.5
1c	50	100.0	68.0
	150	40.0	73.2
	300	13.2	44.3
	600	2.3	21.1
5b	50	75.6	100.0
	150	67.6	89.6
	300	34.9	50.9
	600	5.1	51.5

a DMSO, dimethyl sulfoxide; HMGR, 3-hydroxy-methyl-glutaryl-CoA reductase.

b The dashes mean that the treatment was not applied to the control strains.

### Docking suggests the interaction of the test compounds with HMGR of C. glabrata.

**FIG 4:**
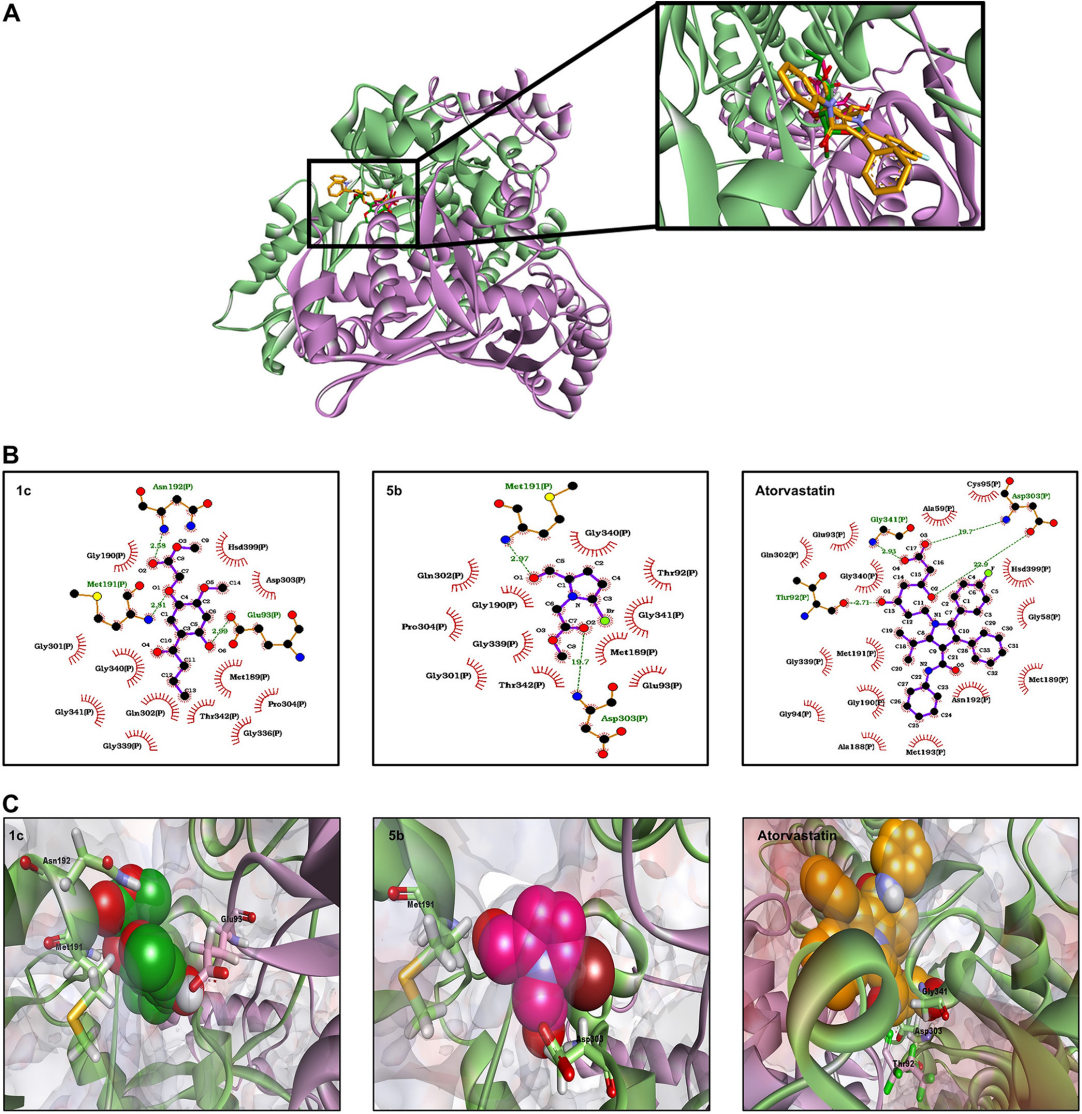
Schematic binding mode of 1c, 5b, and atorvastatin with the catalytic portion of CgHMGR. (A) Structural model of CgHMGR, with subunits a and b corresponding to the catalytic domain colored in green and purple, respectively. A magnified visualization of the ligands interacting with the active site is shown in the black box. (B) Predicted binding mode of 1c, 5b, and atorvastatin with the catalytic portion of CgHMGR. The docking simulation was conducted with AUTODOCK 4. In the two-dimensional model (obtained by using software), electrostatic and van der Waals interactions between the amino acid residues and the compounds are portrayed as red semicircles with rays. Hydrogen bonds are depicted by green dotted lines, and their size is denoted in angstroms. (C) Three-dimensional representation of the docking complexes of CgHMGR with 1c, 5b, and atorvastatin. The α-helix and β-strand structures are depicted as ribbons, colored in blue (subunit a) and purple (subunit b). The molecular surface electrostatic charges are shown. Amino acid residues that interact with ligands through H-bonds are illustrated as sticks, and ligands are spheres. The figure is an original creation designed by J. Ortiz-Álvarez (coauthor of this work) performed with Discovery Studio 2020 Client and LigProt+ software.

Docking simulations displayed the hypothetical interaction of the compounds with CgHMGR. The related values for 1c and 5b are shown in Table 5. 1c has the highest binding energy *in silico*, which correlates with the *in vitro* results (Table 1). Atorvastatin had the lowest binding energy (Table 5). The interaction of 1c and 5b with the amino acid residues in the catalytic site is depicted in Fig. 4. For 1c, hydrogen bonds 2.58 to 2.99 Å in length formed from the hydroxyl groups at C-5 and C-8 to Glu93 and Asn192, respectively, and there was an electrostatic interaction of the O11 methoxy group with Met191. For 5b, hydrogen bonds 2.19 and 19.7 Å in length formed from the hydroxyl groups (at C-5 and Met191) and the carboxyl group (at C-7 and Asp303). The interaction between atorvastatin and the HMGR catalytic site revealed that van der Waals interactions are predominant, although two hydrogen bonds (of 19.7 and 22.7 Å) were detected between the carboxyl group at C-17 and Gly341. Additionally, Asp303 interacted by hydrogen bonds with the carboxyl group at C-17 and the hydroxyl group at C-15 (Fig. 4). The calculated binding energies of 1c and 5b (−5.99 and −5.71 kcal/mol, respectively) were better than those found for α-asarone and atorvastatin (4.53 and −2.13 kcal/mol, respectively) (Table 5).

**TABLE 5:** Docking data results of the binding mode between atorvastatin, 1c, and 5b at the catalytic site of CgHMGR^*a*^

Compound	Binding energy (Kcal/mol)	Interacting residues	Residues with polar interactions	Residues with hydrophobic interactions	References
α-Asarone	−4.53	Glu93, Lys227, Hsd399	Glu93, Lys227, Hsd399		Andrade-Pavon et al. (9)
Atorvastatin	−2.13 ± 1.107	Gly58, Ala59, Thr92, Glu93, Gly94, Ala188, Met189, Gly190, Met191, Asn192, Met193, Gln302, Asp303, Gly339, Gly340, Gly341, Hsd399	Thr92, Asp303, Gly341	Gly58, Ala59, Glu93, Gly94, Ala188, Met189, Gly190, Met191, Asn192, Met193, Gln302, Gly339, Gly340, Hsd399	This work
1c	−5.99 ± 0.104	Glu93, Met189, Gly190, Met191, Asn192, Met189, Gly301, Gln302, Asp303, Pro304, Gly336, Gly339, Gly340, Gly341, Thr342, Hsd399	Glu93, Met191, Asn192	Met189, Gly190, Met189, Gly301, Gln302, Asp303, Pro304, Gly336, Gly339, Gly340, Gly341, Thr342, Hsd399	This work
5b	−5.71 ± 0.004	Thr92, Glu93, Met189, Gly190, Met191, Gly301, Gln302, Asp303, Pro304, Gly339, Gly340, Gly341, Thr342	Met191, Asp303	Thr92, Glu93, Met189, Gly190, Gly301, Gln302, Pro304, Gly339, Gly340, Gly341, Thr342	This work

a CgHMGR, C. glabrata 3-hydroxy-methyl-glutaryl-CoA reductase.

## DISCUSSION

The problem of drug-resistant strains will always exist due to the process of natural evolution and the selection of yeasts and bacteria (14). Therefore, the probability of applying an effective treatment to patients would be increased with the existence of a broad battery of antifungal agents, as well as distinct molecular targets among such drugs.

The HMGR enzyme (particularly CgHMGR) has for some time been proposed as a possible target, leading to the study of some cholesterol-lowering drugs (e.g., simvastatin and atorvastatin) as inhibitors of the growth of pathogenic yeasts (10, 15). According to *in vitro* evolutionary experiments, treatment of C. glabrata with some statins may allow for the selection of mutants. However, gene sequencing has not detected any changes in the catalytic domain of CgHMGR, indicating no effect on HMGR activity. C. glabrata is a useful model for examining resistance to statins and the precise molecular mechanisms of resistance to compounds that inhibit the CgHMGR enzyme (5).

In the current effort, three series of compounds were evaluated as inhibitors of C. glabrata. The two best derivatives were selected to determine their effect on yeast growth and ergosterol synthesis. Complementary studies were carried out with yeast growth rescue assays and docking simulations.

The compounds presently investigated were originally designed as lipid-lowering (11) and anti-inflammatory agents (12). Their chemical structure could plausibly enable them to inhibit the activity of the CgHMGR enzyme. In fact, substituted pyrroles have been considered antifungals (13, 16), and their fungicidal activity is reported. However, the possible molecular target has not been previously explored in an in-depth manner.

Compounds such as statins (e.g., simvastatin and atorvastatin) and fibrates that inhibit HMGR have been administered to lower cholesterol levels in patients (17). Additionally, they have been assessed as growth inhibitors of Candida spp., Aspergillus spp., and Ustilago maydis (7–10, 15, 18). Based on its hypercholesterolemic activity, α-asarone underwent initial studies (19, 20) that resulted in a finding of high toxicity. Thus, new derivative compounds have been designed and synthesized and have produced good activity against different fungi, such as C. glabrata and U. maydis (9, 18).

When the test compounds were examined *in vitro*, the growth inhibition of both strains of C. glabrata was better for 1c than for 5b and α-asarone. On the other hand, 5b did not induce a greater growth inhibition than its reference compound, atorvastatin. The latter statin, bearing a substituted pyrrolic ring, has already been proposed as an antifungal agent to inhibit the growth of Candida spp. (15). Although the antifungal activity of 1c has already been studied (11), this is the first evaluation, to our knowledge, of its effect on an opportunistic pathogenic yeast. Furthermore, the current investigation constitutes the first in-depth exploration of the mechanism of action and molecular target of inhibition by the test compounds.

According to the yeast growth rescue experiment, the test compounds likely inhibited the pathway for sterol biosynthesis (9, 15). The addition of ergosterol to C. glabrata CBS 138 resulted in a recovery of growth at a level below that of the control (without treatment with an inhibitor), while its addition to C. glabrata 43 led to growth that surpassed the control level. This behavior can be explained by what is observed in the fluconazole-resistant C. glabrata strains, in which the consumption and metabolism of sterols might be affected by mutations in the *ERG11* gene. Moreover, the exposure of susceptible C. glabrata strains to fluconazole (an inhibitor of ergosterol synthesis) causes a coordinated action between the consumption and production of ergosterol. Hence, the present test compounds probably inhibit the pathway for sterol biosynthesis, as fluconazole does (21, 22).

Ergosterol is an essential sterol of yeast cell membranes, and deficiencies in sterol biosynthesis cause pleiotropic defects as an adaption to stress. A study was carried out on role of fluconazole in the expression of genes involved in ergosterol synthesis (e.g., *ERG2*, *ERG3*, *ERG4*, *ERG10*, and *ERG11*) and of other genes participating in the regulation of sterol metabolism in fungi. It was concluded that sterol biosynthesis and sterol metabolism act in a coordinated and collaborative manner to support growth and mediate resistance in C. glabrata. This takes place through gene expression dynamics in response to azole treatment and other environmental challenges (22). There are as yet no reports, to our knowledge, on the pleiotropic role played by statins or other compounds that act by inhibiting the HMGR enzyme of C. glabrata or other fungi. Hence, it would be worthwhile to study the effect of inhibitors of HMGR in C. glabrata on the expression of genes involved in resistance to such antifungal agents.

Since 1c and 5b inhibited ergosterol synthesis, they may reduce the activity of the *Cg*HMGR enzyme (15). A better inhibition of the production of ergosterol was found in both strains of C. glabrata for 1c than in its control (α-asarone) or 5b. Of these three compounds, 1c had the lowest IC_50_. Previous publications have documented the capability of simvastatin, α-asarone, and derivatives of the latter to inhibit recombinant CgHMGR (8, 9).

A correlation has been detected in C. albicans strains between their sensitivity to azoles and their total ergosterol concentration (23). Therefore, it was important to demonstrate that the test compounds were capable of inhibiting the synthesis of ergosterol in both strains of C. glabrata (the fluconazole-susceptible and -resistant strains).

The experimental results from the assays on yeast growth inhibition and the inhibition of ergosterol synthesis were complemented by docking simulations based on molecular coupling between the test compounds and CgHMGR. The binding energy values calculated for 1c and 5b were congruent with the *in vitro* findings for these two compounds. 1c exhibited the lowest binding energies and the best *in vitro* inhibition of yeast growth. Better binding to the active site of CgHMGR was displayed by 1c and 5b than α-asarone and its derivatives, based on the calculated binding energies of the present study for the former two and reports in the literature for the latter (9). This supports the *in vitro* results, in which 1c and 5b showed the greatest inhibition of yeast growth and of ergosterol synthesis.

The high binding energy determined from the docking of 1c and 5b into the active site of CgHMGR may stem from the addition of the ester and hydroxyl groups to the molecule, elements that do not exist in the structure of α-asarone. The hydroxyl group of 1c might play a crucial role in the suitable binding mode of the compounds with HMGR (19, 20). Perhaps the chemical structure also confers a strong binding mode, considering the generation of hydrogen bonds with a short distance between atoms. On the other hand, the unsuitable binding mode of atorvastatin with CgHMGR possibly owes itself to steric interference of the chemical structure with a proper approach to the catalytic site of HMGR, as well as to the longer distance of the hydrogen bonds observed in the atorvastatin-CgHMGR complex (19.7 and 22.9 Å), which would confer weaker binding. Actually, atorvastatin has exhibited weak binding energy (−2.89 kcal/mol) with the catalytic site of human HMGR (24), corroborating the present results with atorvastatin and CgHMGR. Interestingly, α-asarone, simvastatin, and the substrate HMG-CoA showed high and almost identical binding energy for the catalytic site of human HMGR (20). Hence, the structural differences between human HMGR and CgHMGR may influence the binding mode.

Molecular modeling of proteins is a useful analytical technique that in the future should allow for the characterization of mutants in the *CgHmgr* gene, a phenotype resistant to antifungal inhibitors of the HMGR enzyme. Such resistance could be explained by changes in the protein related to its tertiary structure or by the capacity of inhibitors to bind with the amino acids of the catalytic site, among other possibilities. Indeed, molecular modeling analysis and mutations in the *ERG11* gene, encoding the enzyme 14α-lanosterol demethylase (CYP51), have already been carried out with distinct C. albicans strains. Thus, a molecular explanation can be provided for the resistance or sensitivity of these strains to different azoles (25).

### Conclusions.

Three series of plausible inhibitors of the CgHMGR enzyme were designed, synthesized, and tested for the inhibition of yeast growth. The two best candidates, 1c (structurally related to fibrates) and 5b (structurally related to atorvastatin), were chosen for further experiments. Compared to 5b, treatment with 1c led to a greater inhibition of yeast growth and ergosterol synthesis. The fact that the target of 1c is the pathway for the synthesis of ergosterol was demonstrated by the decrease it caused in the level of ergosterol, as well as the posterior rescue of yeast viability by the addition of exogenous ergosterol. According to the docking analysis, the present two test compounds display a better binding mode with CgHMGR than α-asarone and atorvastatin, supporting the experimental results.

There are many advantages to the rational design of antifungal compounds that are derived from known drugs (statins, fibrates, etc.), have a defined chemical structure, and are directed at a specific target. Their pharmacokinetics and pharmacodynamics can be inferred, suggesting potential redesign strategies to make them more specific, more potent, and less toxic. Based on the molecular modeling analysis, a plausible interaction of the inhibitor with the target protein is visualized and analyzed, thus providing insights into the possible mechanisms of resistance of a yeast to an antifungal agent. Such resistance might be explained on the basis of changes in the tertiary structure of the protein or in the binding mode of inhibitors with their target. The fibrate-related compound, 1c, herein proved to be a good candidate for further research on its antifungal activity. Modifications of the compound should be considered to achieve greater specificity and potency. The derivatives could then be examined with *in vivo* animal models at a therapeutic dose. Other important areas to be explored are its toxicity and the inhibition of the recombinant CgHMGR enzyme.

## MATERIALS AND METHODS

### Strains and culture media.

C. glabrata CBS 138 and C. glabrata 43 are susceptible and resistant to fluconazole, respectively (9). They were employed to examine the antifungal effect and ergosterol inhibition produced by the current test compounds. C. glabrata CBS 138 was donated by Bernard Dujon of the Pasteur Institute (Paris, France). C. glabrata, C. albicans ATCC 10231, and C. krusei ATCC 14423 strains were stored at –70°C in 50% (vol/vol) anhydrous glycerol (Sigma-Aldrich). They were recovered in yeast extract-peptone-dextrose (YPD) medium (1% yeast extract, 2% casein peptone, and 2% dextrose anhydrous powder; J.T. Baker) at 37°C under orbital shaking at 120 rpm, to be used as inoculum in the assays. The RPMI 1640 medium (Sigma-Aldrich) was prepared in accordance with the standard procedures of the Clinical and Laboratory Standards Institute (CLSI).

### Evaluation of the growth inhibition of C. albicans and C. glabrata.

To identify the compounds with the greatest potential antifungal activity, all the compounds in the three series were examined, together with three reference compounds (fluconazole, α-asarone, and simvastatin), for their effect on the growth of two strains of C. albicans and two strains of C. glabrata. Yeast cells were cultured in slightly stirred YPD medium at 37°C for 24 h and were later adjusted to a density of 0.5 (As_620_) to obtain a new inoculum. A stock solution, prepared with dimethyl sulfoxide (DMSO) and 10 mM each of the inhibitors, was added (50 μL) in a petri dish to afford a final inhibitor concentration of 50, 300, or 600 μM. Subsequently, YPD medium (25 mL) was added, and the mixture was slightly stirred until a homogenous solid was formed. The solidified media were inoculated with 20 μL of each of the Candida strains (previously adjusted) in a section of the petri dish and then incubated at 37°C for 24 h (18).

To best select the possible antifungal compounds, spot dilution susceptibility assays were performed with the *Candida* spp. strains. Cells grown in YPD broth and harvested during the logarithmic-phase were adjusted to 2 × 10^7^ cells/mL and then further incubated at 37°C for 24 h in the presence of inhibitors/antifungals at different concentrations (50, 300, or 600 μM) (26). After treatment, a spot dilution test was carried out as described by Reséndiz-Sánchez et al. (26). Serial 10-fold dilutions were prepared, with 5 μL of each dilution spotted onto a YPD agar plate and incubated on solid YPD medium at 37°C for 24 h. Based on the results observed after the final incubation, two inhibitors were selected for further experiments: 1c from the fibrate derivatives and 5b from the substituted pyrroles.

### *In vitro* activity of the synthetic compounds against *Candida* spp.

The effect of 1c and 5b on the growth of C. glabrata CBS 138 and C. glabrata 43 was evaluated by using the CLSI M27-A3 microdilution method. Briefly, stock solutions of antifungal compounds were prepared, from which the experimental concentrations were obtained in RPMI 1640 medium (Sigma-Aldrich). Fluconazole, simvastatin, atorvastatin, and α-asarone served as reference compounds for examining susceptibility. C. albicans ATCC 10231 and C. krusei ATCC 14423 were the controls for sensitivity and resistance, respectively. The synthetic compounds were dissolved in DMSO immediately before being placed on the microplates and were subsequently incubated at 37°C for 24 h. To avoid an inhibitory effect by the solvent, its volume was less than 10% of the total volume. Growth was quantified by optical density in a Thermo Scientific Multiskan FC microplate spectrophotometer at 620 nm. The values of yeast growth are expressed as the averages of three independent assays.

### C. glabrata growth rescue.

To verify that inhibitors affect yeast viability by inhibiting ergosterol synthesis, a growth rescue experiment was conducted. Growth was first stopped by subjecting yeasts to the sublethal concentration (IC_70–90_) of one of the inhibitors, determined by the CLSI M27-A3 protocol (see section 2.3), and then ergosterol was added. Briefly, to each well of 96-well microplates was added 100 µL of one of the antifungal solutions (2×) prepared in RPMI 1640 medium (Sigma-Aldrich), followed by 80 µL of a yeast suspension adjusted to 1 to 5 × 10^6^ UFC/mL and diluted 1:1,000 with RPMI 1640 medium (Sigma-Aldrich). A stock solution of ergosterol (Sigma-Aldrich) was prepared by dissolving 120 µg/mL in Tween 80/ethanol (1:1) (Sigma-Aldrich). Afterwards, 20 µL of this solution was added to each well, resulting in a final concentration of 12 µg/mL of ergosterol. The controls were yeasts cultured with the vehicle only (in the absence of an inhibitor, the growth control) and those with an inhibitor but without sterol (the growth rescue control) (9, 15, 27).

### Statistical analysis.

The data are expressed as the means of three replicates ± SD. Differences between groups were examined with two-way analysis of variance (ANOVA), with the Bonferroni correction and a 95% confidence interval. Statistical analyses were performed and graphs constructed with GraphPad Prism 5.0, considering statistical significance at *P* < 0.001.

### Ergosterol quantification.

Total sterols were extracted with a slightly modified version of the methodology reported by Arthington-Skaggs et al. (23). Briefly, C. glabrata yeasts were grown in YPD medium by incubation at 37°C for 24 h under constant agitation at 200 rpm. The cell culture was prepared by adjusting it to a optical density of 0.3 (As_620_) in different flasks containing 5 mL of YPD medium, followed by the addition of DMSO solvent (the vehicle) as the control (Sigma-Aldrich, USA) or one of the inhibitors (simvastatin, α-asarone, 1c, or 5b at 50, 150, 300, or 600 μM). For each treatment, the yeasts were incubated at 37°C for 18 h under constant shaking at 200 rpm. The cells were harvested by centrifugation and washed with distilled water. The extraction of sterols was carried out by adjusting each tube to 100 mg of cells for each sample. After establishing the net weight of the pellet, it was mixed with 3 mL of an alcoholic solution of potassium hydroxide (25 g of KOH and 35 mL of distilled water, brought to 100 mL with absolute ethanol) in a vortex for 1 min to extract the sterols (18, 23, 28). The cell suspensions were incubated at 85°C for 1 h, and then the sterols were extracted with a mixture of 1 mL of sterile distilled water and 3 mL of *n*-heptane by vigorously mixing the solution in a vortex for 3 min. The *n*-heptane layer was spectrophotometrically scanned between 230 and 300 nm (BioSpectrometer, Eppendorf). The presence of ergosterol (As_281_._5_ peak) and 24 (28) dihydroxy-ergosterol (24 [28] DHE), a late intermediate (As_230_ peak), can be appreciated by the characteristic four-peaked spectrum indicating sterol absorption. The technique is also capable of revealing a decrease in the level of ergosterol. The absence of detectable levels is evidenced by a flattening of the curve (18, 23, 28).

### Docking of the test compounds on CgHMGR.

The hypothetical three-dimensional structure of CgHMGR was obtained by homology modeling with MODELLER 9.13 software (29), using the crystallographic structure of human HMGR as the template (PDB entry 1DQ8). The quality of the resulting model was evaluated by determining the stereochemical restrictions with a Ramachandran plot constructed on Procheck version 3.5.4 (30). The structure was energetically minimized and equilibrated through molecular dynamic simulations on the NAMD2 program (31), which were performed in 2,000,000 steps for a total run time of 1 ns. The three-dimensional structure of the ligands, obtained with the ChemSketch program (www.acdlabs.com), was subjected to energy optimization and minimization with AVOGADRO software (32). Docking simulations were conducted on AUTODOCK 4 (33), employing the parameters established by Andrade-Pavón et al. (9). Docking results were computed based on a total of 100 runs and 1,250,000,000 generations, analyzed in AutodockTools, and visualized on LigProt+ software (34).

### Synthesis of the compounds tested as potential antifungal agents.

**FIG 5:**
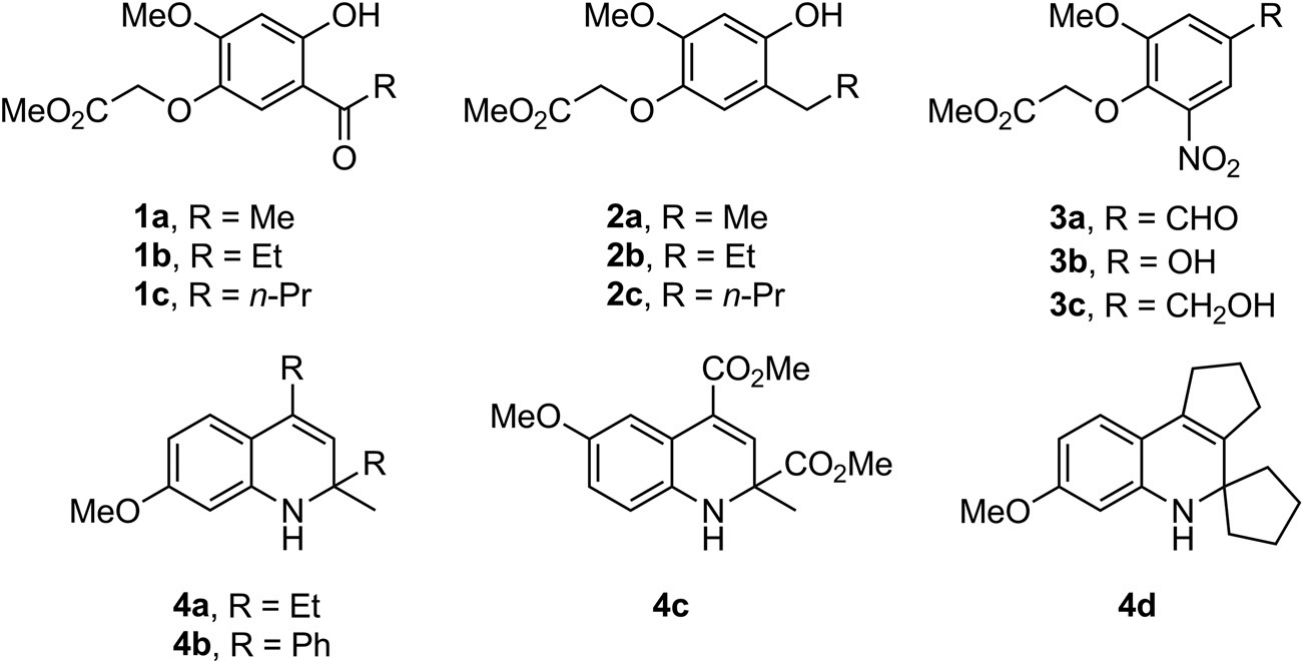
Structures of the fibrate-based analogues 1a through 1c, 2a through 2c, and 3a through 3c (series 1) and 1,2-dihydroquinolines 4a through 4d (series 2) (11).

**FIG 6:**
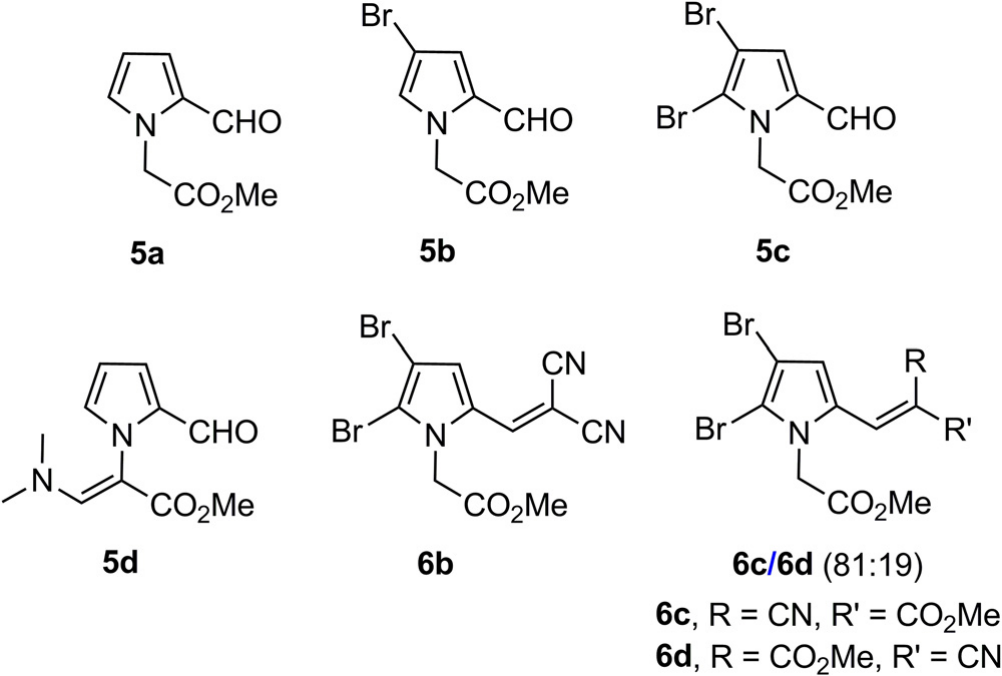
Structures of the substituted pyrroles 5a through 5d and 6b through 6d (series 3) (12).

The fibrate-based derivatives 1a through 1c, 2a through 2c, and 3a through 3c along with 1,2-dihydroquinolines 4a through 4d constituted the first two series of compounds (11) (Fig. 5). The substituted pyrrole derivatives comprised the third series: 5a through 5d and 6b through 6d (12) (Fig. 6). The brominated pyrroles 5b, 5c, and 6b through 6d were designed because of their similarity to some pyrrole-based marine alkaloids known to exert both antifungal and antibacterial activity (13, 16, 35).

### Synthesis of bromopyrroles 5b and 5c.

**SCHEME 1:**
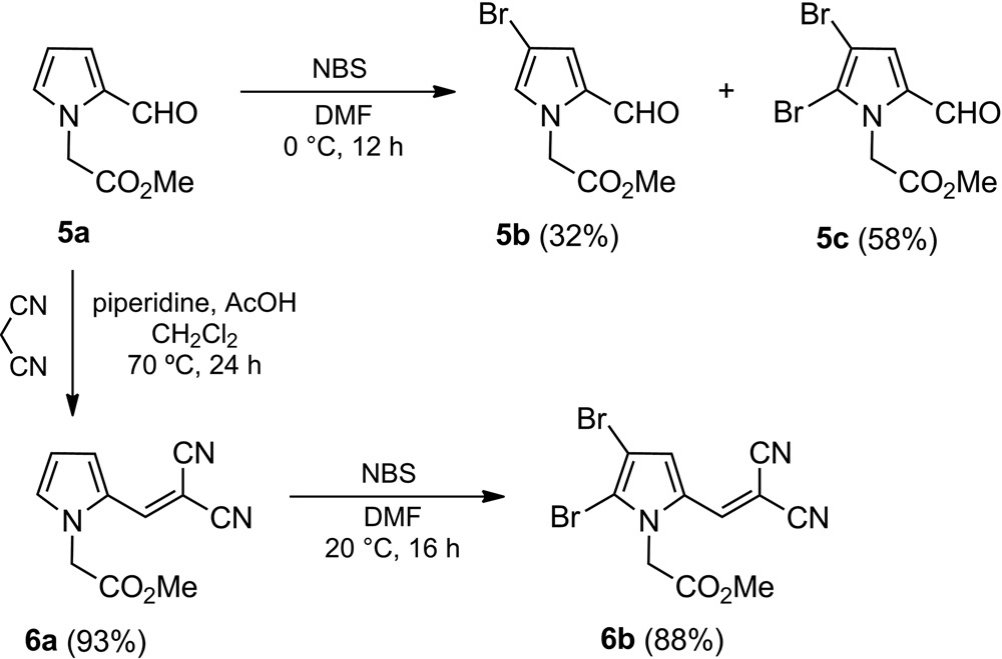
Synthesis of 4-bromopyrroles 5b, 5c, and 6b from pyrrole 5a.

The synthesis of 5a, 5d, 6c, and 6d has been previously reported (11, 12). The preparation of bromopyrroles 5b and 5c was achieved by treatment of compound 5a (12) with *N*-bromosuccinimide (NBS) as the brominating agent under mild reaction conditions (Scheme 1). Even though 1.0 mol equivalent of NBS was employed, a mixture of bromopyrroles 5b and 5c was obtained. Because they were easily separated by column chromatography, an excess of NBS (2.5 mol equivalent) was added to the reaction mixture to give 5b and 5c in 32 and 58% yields, respectively.

The synthesis of dibromopyrrole 6b was carried out by a two-step methodology. The first step consisted of a Knoevenagel reaction of 5a with malononitrile under acid conditions (12) to provide 6a in high yield (Scheme 1). Bromination of the latter with NBS (2.0 mol equivalent) in dimethylformamide (DMF) as the solvent led to the desired product 6b in good yield (88%) (Scheme 1).

### General information.

The melting points were determined on a Krüss KSP 1N capillary melting point apparatus. Infrared (IR) spectra (ATR-FT or KBr) were recorded on a PerkinElmer 2000 spectrophotometer. ^1^H and ^13^C nuclear magnetic resonance (NMRs) spectra were captured on a Varian Mercury (300 MHz) instrument, with CDCl_3_ as the solvent and tetramethylsilane (TMS) as the internal standard. Signal assignments were based on two-dimensional NMR spectra (heteronuclear multiple quantum correlation [HMQC] and heteronuclear multiple-bond correlation [HMBC]). High-resolution mass spectra (HRMS) were obtained (in electron impact mode) on a Jeol JSM-GCMateII spectrometer. Analytical thin-layer chromatography was carried out using E. Merck silica gel 60 F254-coated 0.25 plates, visualized by using a long- and short-wavelength UV lamp. Flash column chromatography was conducted over Natland International Co. silica gel (230-400 and 230-400 mesh). All air moisture-sensitive reactions were carried out under N_2_ using oven-dried glassware. CH_2_Cl_2_ and DMF (Sigma-Aldrich) were distilled over CaH_2_ (Sigma-Aldrich) prior to use. All other reagents (Sigma-Aldrich) were employed without further purification.

### Synthesis of bromopyrroles 5b and 5c.

For methyl 2-(4-bromo-2-formyl-1H-pyrrol-1-yl)acetate (5b) and methyl 2-(2,3-dibromo-5-formyl-1H-pyrrol-1-yl)acetate (5c), a solution of NBS (0.267 g, 1.50 mmol) in anhydrous DMF (2 mL) was added dropwise to a solution of 5a (0.100 g, 0.60 mmol) in anhydrous DMF (5 mL) at 0°C under constant stirring. Stirring continued at 0°C for 12 h. A mixture of water/hexane/EtOAc (1:0.5:0.5) (20 mL) was added, the organic layer dried (Na_2_SO_4_), and the solvent was removed under vacuum. The residue was purified by column chromatography over silica gel (30 g/g crude, hexane/EtOAc, 9:1) leading to 5b (0.062 g, 32%) as a yellow solid and 5c (0.112 g, 58%) as a yellow oil.

The data for 5b are as follows: R*f* 0.43 (hexane/EtOAc, 7:3); mp 203–205°C. IR (film): ῡ 3,121, 2,954, 1,754, 1,666, 1,392, 1,365, 1,219, 1,092, 923, 771 cm^−1^. ^1^H NMR (300 MHz, CDCl_3_): δ 3.78 (s, 3H, CO_2_C*H*_3_), 5.03 (s, 2H, C*H*_2_), 6.92 (br dd, *J *=* *1.8, 1.2 Hz, 1H, H-5′), 6.98 (d, *J *=* *1.8 Hz, 1H, H-3′), 9.47 (d, *J *=* *0.9 Hz, 1H, C*H*O). ^13^C NMR (75.4 MHz, CDCl_3_): δ 50.1 (*C*H_2_), 52.7 (CO_2_*C*H_3_), 97.5 (C-4′), 125.2 (C-3′), 131.2 (C-5′), 131.6 (C-2′), 168.2 (*C*O_2_CH_3_), 179.3 (*C*HO). HRMS (EI): *m*/*z* [M^+^] calculated for C_8_H_8_BrNO_3_: 244.9688; found: 244.9690.

The data for 5c are as follows: R*f* 0.69 (hexane/EtOAc, 7:3); IR (film): ῡ 2,955, 1,755, 1,668, 1,450, 1,397, 1,363, 1,218, 1,005, 810, 776 cm^−1^. ^1^H NMR (300 MHz, CDCl_3_): δ 3.78 (s, 3H, CO_2_C*H*_3_), 5.25 (s, 2H, C*H*_2_), 7.05 (s, 1H, H-4′), 9.32 (s, 1H, C*H*O). ^13^C NMR (75.4 MHz, CDCl_3_): δ 49.0 (*C*H_2_), 52.7 (CO_2_*C*H_3_), 101.1 (C-3′), 118.6 (C-5′), 125.3 (C-4′), 132.4 (C-2′), 167.5 (*C*O_2_CH_3_), 178.2 (*C*HO). HRMS (EI): *m*/*z* [M^+^] calculated for C_8_H_7_Br_2_NO_3_: 322.8793; found: 322.8791.

### Synthesis of pyrroles 6a and 6b.

For methyl 2-(2-(2,2-dicyanovinyl)-1H-pyrrol-1-yl)acetate (6a), in a threaded ACE glass pressure tube with a sealed Teflon screw cap and magnetic stirring bar, a solution of 5a (0.100 g, 0.60 mmol), malononitrile (0.044 g, 0.66 mmol), piperidine (0.026 g, 0.30 mmol), and glacial AcOH (0.029 g, 0.48 mmol) in anhydrous CH_2_Cl_2_ (5 mL) was heated at 70°C for 24 h. The reaction mixture was diluted with CH_2_Cl_2_ (50 mL) and washed with water (25 mL) and an aqueous saturated solution of NaHCO_3_ until neutral. The organic layer was dried (Na_2_SO_4_), and the solvent was removed under vacuum. The residue was purified by column chromatography over silica gel (20 g/g crude, hexane/EtOAc, 9:1) to afford 6a (0.12 g, 93%) as a yellow solid. R*f* 0.51 (hexane/EtOAc, 8:2); mp 203–205°C. IR (KBr): ῡ 3,132, 2,992, 2,220, 1,751, 1,583, 1,476, 1,399, 1,350, 1,328, 1,239, 1,169, 1,132, 1,088, 994, 758, 732 cm^−1^. ^1^H NMR (300 MHz, CDCl_3_): δ 3.83 (s, 3H, CO_2_C*H*_3_), 4.80 (s, 2H, C*H*_2_CO_2_Me), 6.49 (ddd, *J = *4.5, 2.4, 0.6 Hz, 1H, H-4′), 7.10 (dd, *J *=* *2.4, 1.5 Hz, 1H, H-5′), 7.38 (s, 1H, H-1′′), 7.73 (ddd, *J *=* *4.5, 1.5, 0.6 Hz, 1H, H-3′). ^13^C NMR (75.4 MHz, CDCl_3_): δ 48.3 (*C*H_2_CO_2_Me), 53.3 (CO_2_*C*H_3_), 72.5 (C-2 ″), 113.4 (C-4′), 114.0 (*C*N), 115.3 (*C*N), 121.1 (C-3′), 127.2 (C-2′), 131.6 (C-5′). 142.7 (C-1 ″), 167.3 (*C*O_2_CH_3_). HRMS (EI): *m*/*z* [M^+^] calculated for C_12_H_9_N_3_O_2_: 215.0695; found: 215.0694.

For methyl 2-(2,3-dibromo-5-(2,2-dicyanovinyl)-1H-pyrrol-1-yl)acetate (6b), to a solution of 6a (0.100 g, 0.47 mmol) in anhydrous DMF (3 mL) at 0°C under constant stirring, a solution of NBS (0.166 g, 0.93 mmol) in anhydrous DMF (3 mL) was added dropwise. Stirring continued at 20°C for 16 h. A mixture of water/hexane/EtOAc (0.5:1:1) (30 mL) was added, the organic layer dried (Na_2_SO_4_), and the solvent was removed under vacuum. The residue was purified by column chromatography over silica gel (20 g/g crude, hexane/EtOAc, 7:3) to give 6b (0.25 g, 88%) as a yellow solid. R*f* 0.44 (hexane/EtOAc, 8:2); mp 267–269°C. IR (KBr): ῡ 3,004, 2,956, 2,223, 1,741, 1,583, 1,420, 1,391, 1,339, 1,243, 1,167, 1,125, 1,004, 983, 815, 738, 687 cm^−1^. ^1^H NMR (300 MHz, CDCl_3_): δ 3.85 (s, 3H, CO_2_C*H*_3_), 4.89 (s, 2H, C*H*_2_CO_2_Me), 7.31 (d, *J = *0.3 Hz, 1H, H-1′′), 7.76 (d, *J = *0.3 Hz, 1H, H-4′). ^13^C NMR (75.4 MHz, CDCl_3_): δ 47.8 (*C*H_2_CO_2_Me), 53.5 (CO_2_*C*H_3_), 75.1 (C-2”), 104.8 (C-3′), 113.3 (*C*N), 114.4 (*C*N), 118.6 (C-5′), 121.5 (C-4′), 128.3 (C-2′), 141.4 (C-1′′), 166.1 (*C*O_2_CH_3_). HRMS (EI): *m*/*z* [M^+^] calculated for C_11_H_7_Br_2_N_3_O_2_: 370.8905; found: 370.8905.

### Reference compounds for the tests of the three series of potential antifungal compounds 1a through 1c, 2a through 2c, 3a through 3c, 4a through 4d, 5a through 5d, and 6b through 6d.

Depending on the experiment, different inhibitors served as the reference compounds. In the case of the sensitivity tests and docking analysis, α-asarone was the control for the fibrate-based derivatives 1a through 1c, 2a through 2c, and 3a through 3c (series 1) along with 1,2-dihydroquinolines 4a through 4d (series 2), and atorvastatin served this purpose for the substituted pyrroles 5a through 5d and 6b through 6d (series 3). In the experiment to determine the effect of the compounds on the biosynthesis of ergosterol, simvastatin and α-asarone were employed. It has been reported that these two compounds are capable of inhibiting recombinant Cg-HMGR, thus affecting the production of ergosterol (9).
