# Supplementary material for: Antifungal Activity of Fibrate-Based Compounds and Substituted Pyrroles That Inhibit the Enzyme 3-Hydroxy-methyl-glutaryl-CoA Reductase of Candida glabrata (CgHMGR), Thus Decreasing Yeast Viability and Ergosterol Synthesis
Source: Microbiol Spectr. 2022 Apr 4;10(2):e01642-21. doi: 10.1128/spectrum.01642-21 (PMC9045318; doi:10.1128/spectrum.01642-21)
Supplement: SUPPLEMENTAL FILE 1 — Supplemental material. Download SPECTRUM01642-21_Supp_1_seq13.pdf, PDF file, 0.3 MB [file spectrum01642-21_supp_1_seq13.pdf]

SUPPLEMENTAL MATERIAL

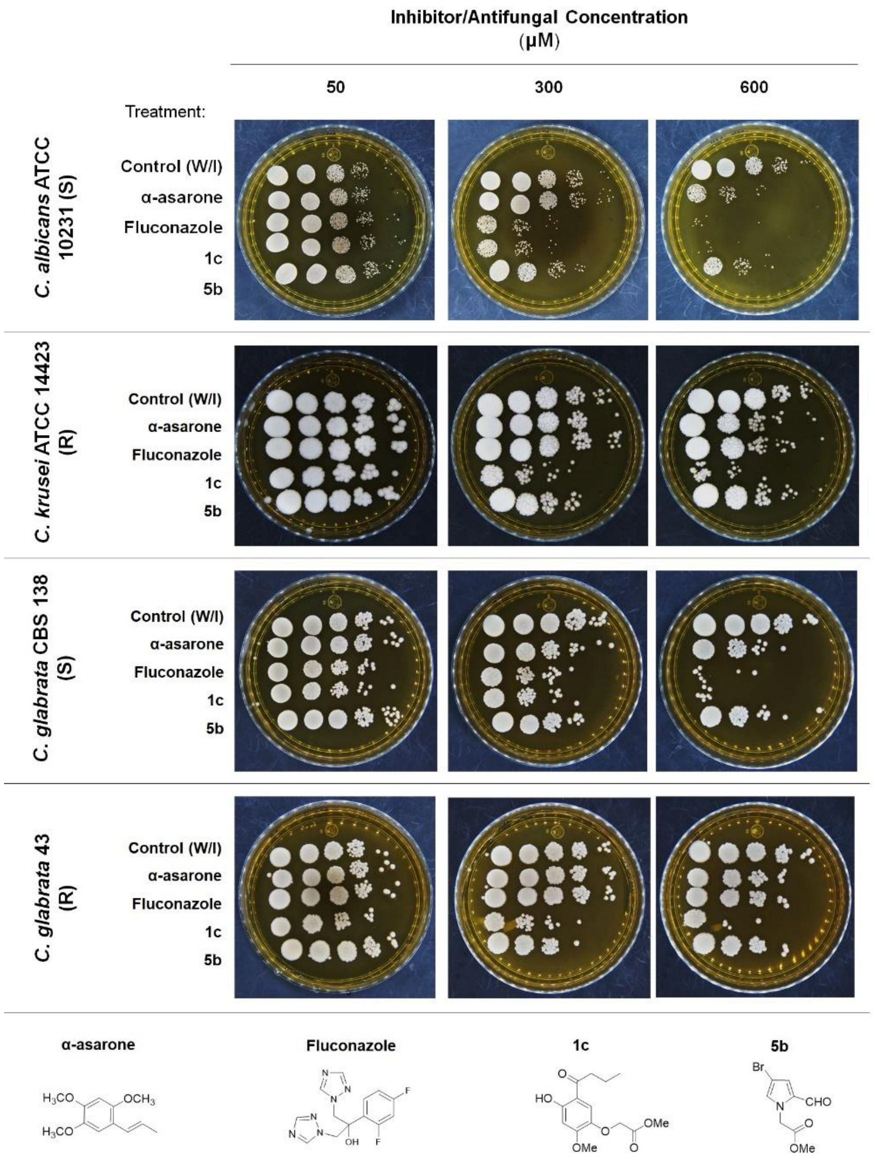

Supplementary Figure 1. Effect of the inhibitors **1c** and **5b** on the growth of *C. glabrata* (CBS138, CGL43), using *C. albicans* ATCC 10231 and *C. krusei* ATCC 14423 as controls. Cells grown in YPD broth and harvested during the logarithmic-phase were adjusted to 2×10^7 cells/mL and then further incubated at 37 ºC for 24 h in the presence of inhibitors/antifungals at different concentrations (50, 300, or 600 μM). After treatment, serial 10-fold dilutions were prepared, with 5 μL of each dilution spotted onto a YPD agar plate and incubated on solid YPD medium at 37 ° C for 24 h. Subsequently, the results were observed.

Supplementary Table 1

The IC_{50} (μM) is given for each compound, being the concentration that inhibits 50% of the ergosterol in *C. glabrata*.

Inhibitor	*C. glabrata* CBS 138	*C. glabrata* 43
**Simvastatin**	67.3	>600
**α -asarone**	117.8	392.5
**1c**	125.1	269.3
**5b**	230.3	>600
